# Non-enzymatic Glucose Detection Mechanism on Pt: A Surface Interrogation Scanning Electrochemical Microscopy Investigation

**DOI:** 10.1021/acselectrochem.5c00464

**Published:** 2026-04-27

**Authors:** Nazario Martino, Francesco Panico, Frank Marken, Alberto Vertova, Alessandro Minguzzi

**Affiliations:** † Dipartimento di Chimica, 9304 Università degli Studi di Milano, via Golgi 19, 20133 Milano, Italy; ‡ Department of Chemistry, 1555 University of Bath, BA2 7AY Bath, U.K.; § Consorzio Interuniversitario di Scienze e Tecnologia dei Materiali, Via San Giusti 9, 50121 Firenze, Italy; ∥ Dipartimento di Energia, Politecnico di Milano, Via Lambruschini 4a, 20156 Milano, Italy

**Keywords:** glucose electrooxidation, platinum, OH_ads_, PtOx, surface interrogation SECM, non-enzymatic glucose sensing

## Abstract

Understanding the surface-state dependence of glucose electrooxidation on platinum is essential for advancing non-enzymatic glucose sensing. Mechanistic assignment based on classical cyclic voltammetry (CV) remains ambiguous because CV convolves glucose adsorption-dehydrogenation with the simultaneous formation and removal of Pt-oxygenated species. Here, we investigate glucose electrooxidation on polycrystalline Pt in phosphate buffer solution (pH 7.3) by combining conventional voltammetry with surface interrogation scanning electrochemical microscopy (SI-SECM), which enables quantitative titration of surface-bound oxidizing equivalents. Using the [Ru­(NH_3_)_6_]^3+/2+^ redox couple as a mediator, the Pt substrate was pre-polarized for 30 s at controlled potentials, then switched to open circuit while tip voltammetry generated Ru^2+^ to titrate oxidising species (PtOx_ads_) formed on the substrate. SI-SECM reveals that titratable PtOx_ads_ begins to form at ∼0.61 V vs RHE and increases with the increase of the pre-polarizing potential. Results evidence how, in the 0.61–0.81 V potential range, commonly associated with incipient hydroxide/OH, these oxidizing species are not consumed by glucose. In contrast, at more anodic potentials (≥0.9 V), the PtOx_ads_ amount is significantly reduced in the presence of glucose, indicating direct involvement of higher-potential Pt-oxide species in glucose oxidation. These outcomes clarify the potential-dependent reactivity of Pt-oxygenated layers in neutral media and demonstrate SI-SECM as a powerful approach to decoupling and quantifying surface oxidants that cannot be resolved by CV alone.

## Graphical abstract

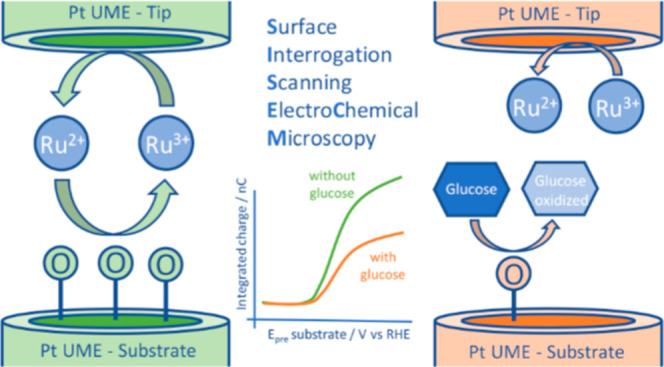

## 1 Introduction

Diabetes is a chronic condition that arises either when the pancreas does not produce sufficient insulin or when the body cannot effectively use the insulin it produces.^1^ It has been identified by the World Health Organization (WHO) as one of the four major non-communicable diseases. Moreover, adults with diabetes in low-income countries experience higher rates of mortality and morbidity even among those with access to universal healthcare systems and the quality of diabetes care varies substantially across countries.^2^ The number of diabetes is expected to increase up to 783 million by 2045 as the International Diabetes Federation (IDF) expectation, making diabetes the seventh-leading cause of mortality.^2–4^ Diabetes, being a chronic disease, can result in several comorbidities, including kidney disease, vision impairment, strokes, and retinopathy.^5^ Effective diabetes management, supported by blood sugar monitoring devices, is crucial for improving quality of life and preventing complications in both type 1 and type 2 diabetes.^1,3,4,6–8^ These devices can detect glucose within the concentration range typical of blood (4–10 mM) and are applicable to both type 1 and type 2 diabetes patients. In addition, they are particularly user-friendly for individuals who fear fingerstick testing.^3,4^ Among blood glucose monitoring devices, continuous glucose monitoring (CGM) sensors provide 24 h, real-time glucose tracking and are therefore gradually becoming a standard of care in diabetes management.^9^ The efforts over the last decade toward wearable non-invasive glucose monitoring devices have given diabetic patient the hope of painless glucose measurements and disease management.^3^ Currently, most commercial blood glucose sensors rely on enzyme-based electrochemical sensors.^4,10^ The enzymes most frequently employed in these devices are glucose oxidase (GOx) and glucose dehydrogenase (GDH),^4^ the first being particularly valued for its high specificity to glucose and superior stability compared to other enzymes, with different well-known weaknesses.^4^ The inherent drawbacks of enzyme-based biosensors, such as limited stability, high enzyme costs, sterility requirements, and susceptibility to environmental factors, have driven the development of non-enzymatic (catalytic) amperometric sensors. These sensors offer advantages including simplicity, higher sensitivity, and lower cost and have attracted considerable research interest. However, low tolerance to poisoning species remains a major challenge.^11^

In recent years, research has focused on developing novel electrocatalytic materials, that can be effectively characterized also as powders,^12^ such as silver, platinum, gold, nickel, copper, cobalt, iron, and metal–organic frameworks.^13^

Beyond electrochemical methods, the future of glucose monitoring is increasingly shifting toward non-invasive and minimally invasive technologies.^14–16^ Sweat, saliva, and tear-based glucose sensors^17–19^ are also being explored, offering alternative bio-fluids for glucose detection, but their accuracy and correlation with blood glucose levels require further validation. Microneedle-based interstitial fluid sensors have emerged as a promising alternative, offering continuous glucose monitoring with minimal discomfort.^20^

One of the main goals pursued by many researchers is the electrochemical detection of glucose without the use of enzymes.^21^ Non-enzymatic glucose sensors are steadily moving closer to practical application, thanks to the research on new materials and a better understanding of glucose oxidation mechanisms.^6,13^ Platinum, which is discussed in the present work, is among the most commonly used electrocatalytic electrode materials due to its outstanding catalytic activity and good stability.^4^ Moreover, to investigate the oxidation mechanism of glucose on Pt can be profitable also for the developing of abiotic glucose fuel cells, AGFCs, a fuel cell that, oxidizing glucose, can produce electrical energy.^22–24^ Directly using glucose without fermentation or reforming can save energy by streamlining the overall conversion process. Moreover, glucose is non-toxic, non-flammable, low-cost, and easy to handle. However, its direct oxidation on metal electrodes proceeds slowly at room temperature and requires highly active catalysts to efficiently generate electricity; particularly, Pt is largely studied. Finally, AGFCs work better in the alkaline environment.^25^

Coming back to sensors, the electrocatalytic process typically occurs through the adsorption of the analyte onto the electrode surface; the adsorption energy should be neither too high nor too low.^26^ Moreover, the behaviour of an electrocatalytic material can be deeply affected by the support,^27^ and it can be influenced by changes in the metal oxidation state, which may modify the adsorbate-metal interaction and promote product desorption.^28^ The geometry of the electrode plays a crucial role in catalytic processes, as demonstrated by single-crystal studies of glucose oxidation. Pletcher^26^ proposed a concerted mechanism where hydrogen abstraction and analyte adsorption occur simultaneously. In glucose electrooxidation, the rate-determining step is hemiacetalic hydrogen removal, coinciding with chemisorption, meaning that adjacent metal sites are occupied by a single adsorbate. However, active metal centers alone do not explain the oxidative role of hydroxide anions adsorbed onto the Pt surface. Studies show glucose and other organic electrooxidation align with the onset of adsorbed hydroxide species (OH_ads_).^4,28^ Burke emphasized the role of the hydrous oxide layer, proposing the “Incipient Hydrous Oxide Adatom Mediator” (IHOAM) model.^28–30^ This theory is based on the idea that metallic “active” surface atoms undergo a premonolayer oxidation phase, forming a reactive incipient hydrous oxide layer (OH_ads_) that facilitates organic molecule oxidation. This is particularly relevant for platinum electrodes, which have been extensively studied in various reactions, including glucose oxidation, under acidic, alkaline, and neutral conditions.^6,13,31,32^ However, direct evidence confirming the involvement of Pt-oxidation intermediates in glucose oxidation remains elusive. As reported by Rodriguez et al.,^33^ scanning electrochemical microscopy (SECM) is a powerful analytical technique for investigating material surfaces. It employs an (ultra)­microelectrode (UME) that can be precisely positioned across the electrochemical cell to perform measurements at very small distances—from micrometers down to nanometers from the surface/electrode under study. In surface interrogation SECM (SI-SECM), a redox mediator in solution is used to “titrate” adsorbed oxidizing species, enabling the selective detection and quantification of reactive intermediates formed during substrate electrode operation. First, the substrate electrode is pulsed or scanned to a potential that induces oxidation, leading to the formation of an oxidizing adsorbed species, PtOx_ads_. The substrate is then switched to an open circuit while the tip potential is scanned to reduce a mediator (O_M_ + e^–^ → *R*_M_). The mediator reduced species, *R*_M_, diffuses through the tip-substrate gap, reaches the adsorbate PtOx_ads_, and reacts with it, regenerating O_M_ while converting PtOx_ads_ into non-oxidizing product. As PtOx_ads_ species are consumed or not active, the system moves to a negative feedback state.^33–35^

The aim of this study is to verify the IHOAM mechanism proposed by Burke and Pletcher using SI-SECM. In this study, the surface of a platinum microelectrode, working as the substrate, is polarized progressively to potential where an oxide/incipient hydroxide layer is formed, in the absence and in the presence of glucose, that can be subjected to oxidation. Ru^3+^/Ru^2+^ couple has been used as the mediator for the titration of platinum oxidizing species formed on the substrate surface, as a function of polarizing potential (see Figure 1).

**Figure 1:**
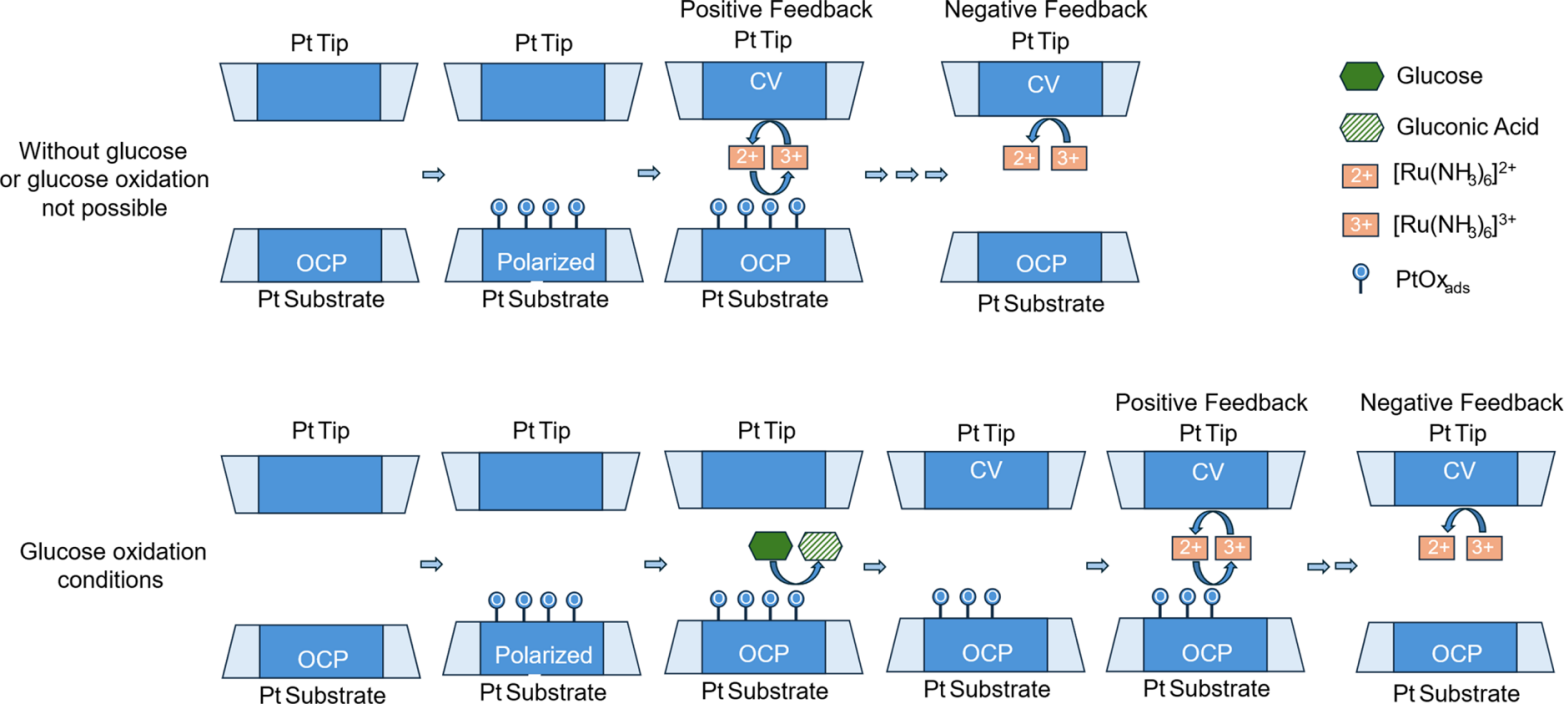
SI-SECM working scheme for the experiment proposed in this work. Glu: glucose, Glu acid: gluconic acid, Ru^2+^/Ru^3+^: ruthenium ions acting as the redox mediator, O: oxidizing species (PtOx_ads_) layer on the platinum electrode, OCP: open-circuit potential, polarized: electrode polarized at a definite potential, CV: cyclic voltammetry.

This allows, in principle, determination of the amount of PtOx_ads_ modulated by glucose oxidation. In the absence of glucose, after the substrate was polarized at the selected constant potential to form PtOx_ads_, the Ru^3+^ to Ru^2+^ reaction on the Pt tip allowed for the titration of PtOx_ads_ amount, with positive feedback in Figure 1. In the presence of glucose, CVs on the tip allowed us to study the glucose electrooxidation (EO) mechanism in neutral pH, in terms of both the influence of substrate constant pre-polarization potential, which determines the amount of PtOx_ads_, and of glucose chemisorption on the subsequent oxidation steps. Moreover, electrochemical characterization revealed how glucose adsorption in the hydrogen region affects the glucose oxidation in the double layer and Pt oxide regions.

## 2 Experimental Section

Experiments were performed in both a 0.1 M phosphate buffer solution (PBS—this acronym is used throughout the entire manuscript) at pH 7.3 (K_2_HPO_4_ and KHPO_4_, Sigma-Aldrich, purity >99.0%), prepared with ultrapure water (Milli-Q, 18,2 MΩ/cm), and in a 0.5 M glucose solution (D-(+)-glucose, minimum 99.5%, Sigma), prepared using the previously described PBS. For SI-SECM measurements, also 1 mM Ru­(NH_3_)_6_Cl_3_ (Sigma-Aldrich 98%) in PBS was added as the mediator.

### 2.1 Pt Macro-Electrode Characterization

These measurements have been performed using a classical 3 electrode setup in a single compartment electrochemical cell: a platinum disk electrode (Pt-DE) tip (a RDE tip of 5.0 mm diameter used statically) was used as the working electrode, and a Pt wire and a Ag | AgCl (3 M KCl) electrode were used as counter electrode (CE) and reference electrodes (RE), respectively, for all the measurements. To avoid chloride contamination, RE was inserted in a double bridge filled with a 3 M KNO_3_ + 3% w/v agar gel solution. Prior to any run, the Pt-DE tip was mechanically polished using sandpaper at different mesh up to 0.05 μm alumina powder on cloth pads (Metkon). Finally, WE was electrochemically cleaned by cycling the potential at 0.5 V·s^–1^ between −0.2 and +1.25 V vs. RE in PBS for 10 cycles. For each run, the electrolytic solution was degassed for 15 min with nitrogen. All of the tests were carried out at 25 °C using a jacketed electrochemical cell.

For sake of clarity, all potentials are referred to the reversible hydrogen electrode, RHE. This conversion has been applied

$$E_{RHE}=E_{ref=Ag/AgCl}+0.059\;pH+0.198$$

after having standardized the Ag reference with RHE.

### 2.2 Pt Epoxy Resin Sheath Ultra-Microelectrode (UME) Preparation

Pt UME were used both as substrate and tip during the below described SI-SECM characterization. The procedure to prepare epoxy resin sheathed-UME (Pt-UME) has been detailed by Zhao et al.;^36^ briefly, a copper wire was connected to a Pt microwire (99.99% purity, Goodfellow, 50 μm diameter) using a conductive gluing agent. After hardening, the microwire and copper wire were covered with epoxy resin using a Teflon tube as a mold. Once dried, the Teflon tube is removed, leaving a rod-shaped plastic envelope terminated with a Pt-microdisk electrode. The shape of the electrode can be adjusted, and the Pt microdisk can be centered by manual polishing. Prior to the measurement, the microdisk is cleaned with 0.05 μm alumina slurry.

The *R*_g_ value, which is defined as the ratio between the radius of the plastic insulator (*r*_INS_) and the radius of the inner Pt wire (*r*_M_), gives information about the quality of the microelectrode. Smaller *R*_g_ values allow us to approach very closely the investigated surface, whereas larger values do not allow for a perfect perpendicular approach, so that a part of the plastic sheath could touch the surface thus hindering the possibility to reach the optimal substrate-tip gap distance for SI-SECM measurements.^37,38^ Measuring *r*_INS_, after the UME preparation (see Figure S1) and determining *r*_M_, by eq 1 and CVs in the presence of a redox couple, is possible to calculate the *R*_g_.

1 $$i_{ss}=4\pi nFD_{x}c_{x}^{*}r_{M}$$

where *i*_ss_ = steady-state current, A; *n* = number of exchanged electrons; *D* = diffusion coefficient of the redox couple, *D* = 8.4 × 10^–6^ cm^2^·s^–1^;^39^ c* = concentration of the redox couple in solution, mol·cm^–3^; and *r*_M_ = radius of the electrode, cm.

For successful SI-SECM characterization, it is crucial that substrate and tip have similar surface area, in term of size and shape, because the tip works both as the titrant generator and detector and the two microelectrodes must approach at a distance comparable to their radius. For this reason, the fabrication of microelectrodes with *R*_g_ values not exceeding 10 is fundamental. The tips used in this study had an estimated *R*_g_ value of 5.

In any case, the effect of *R*_g_ on SECM measurements can be very complex,^40–44^ and it is important also to account for the following aspects such as

- Impact on approach angle
- Effect on tip shielding
- Contribution to hindered diffusion and feedback behavior all well discussed in the proposed literature.

### 2.3 (SI-SECM) Measurements

Experiments were performed using a CHI instrument SECM 920D station (CH Instruments, Austin, TX) and a small cylindrical (5.0 cm diameter) PTFE electrochemical cell. Pt-UME used as the substrate was placed in a drilled hole at the bottom of the cell, then filled with the PBS; Pt-UME used as tip was fixed at the micromotor of the SECM instrument. In the electrochemical cell were placed the CE, a Pt wire, and the RE, Ag | AgCl (3 M KCl) inserted in a double bridge. The cell solution was deaerated using humidified N_2_, to avoid electrolyte solution stripping. Details about the SECM cell and the two Pt-UME alignment procedures, with relevant plots, have been reported in Supporting Information, Chapter 1, Figures S2–S5.

The procedure for operating the SI-SECM measurements was the following. First the substrate was pre-polarized at the selected constant potential for 30 s (*E*_pre_); then, the polarization was stopped and after 2 s of quiet time CVs using the tip as the WE were performed, ranging the potential between +0.63 V and +0.23 V (Ru­(NH_3_)_6_^3+^/Ru­(NH_3_)_6_^2+^ region) at 50 mV·s^–1^, to interrogate the substrate. At the end, the above-described procedure was repeated increasing the *E*_pre_, the substrate constant pre-polarization potential. *E*_pre_ values ranged from +0.01 V to +1.51 V in 100 mV increments (e.g., 0.01 V, 0.11 V, 0.21 V, ...., 1.51 V; positive direction). Then, a second test was carried out, pre-polarizing the Pt substrate at 1.51 V and decreasing the constant pre-polarizing potential in 100 mV decrements from +1.51 V to +0.01 V (e.g., 1.51 V, 1.41 V, 1.31 V, ..., 0.01 V; negative direction).

## 3 Results and Discussion

### 3.1 Preliminary Studies with the Pt Macro-Electrode

Preliminary measurements using Pt-DE have been conducted in order to identify the CV region as described by Pletcher, Toghill, Burke, and Vassilyev^26,28,29,45^ to investigate the phenomena connected with the double-layer region and the oxide region and their influence on the Pt reactivity in the hydrogen region. As can be seen in Figure 2, CVs between 0 and 1.55 V can be divided in three regions according to literature:^26^ (i) hydrogen region (0.12–0.40 V), here chemisorption and dehydrogenation of glucose take place; (ii) double-layer region (0.40–0.85 V), here the oxidation of the chemisorbed glucose should take place, according to Burke theory,^28–30^ thanks to the action of OH_ads_. In accordance with the IHOAM model, formed hydroxide on the Pt surface proves to be a strong oxidant for the adsorbed glucose, so enhancing the electrooxidation reaction; (iii) oxide region (>0.90 V), here the platinum is covered by PtO that is the active part in the glucose oxidation, even if not so active as OH_ads_. For this reason, during the cathodic sweep, when the potential reaches the region of OH_ads_ formation, in the double-layer region, the glucose oxidation proceeds faster, thus cleaning the Pt surface with the increase of the glucose oxidation current.

**Figure 2:**
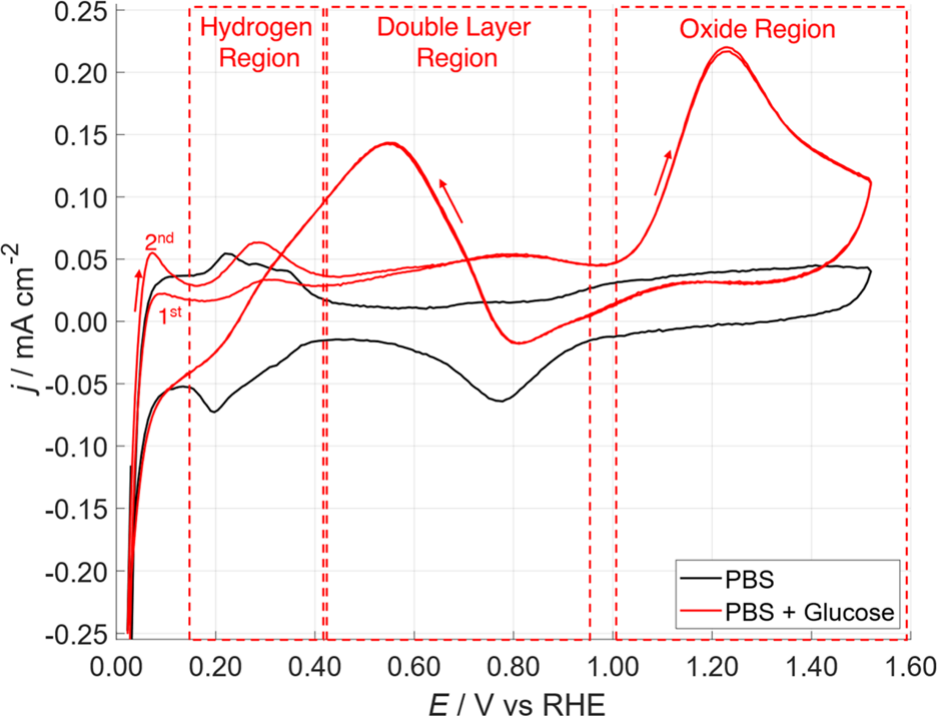
CV of Pt-DE in PBS (black curve) and 0.5 M glucose in PBS (red curve). Starting potential = 0.0 V; two cycles; scan rate 50 mV·s^–1^.

It is notably a significant difference between the first and second cycle in Figure 2, when glucose was present (red curve); the peak in the hydrogen region was higher in the second cycle with respect to the first one. When fresh Pt-DE was immersed in the electrolyte solution, glucose was adsorbed on the Pt surface, thus leading to less availability of the active site; sweeping the electrode potential in the double-layer region, during the cathodic sweep of the first cycle, all the adsorbed glucose can be oxidized (OH_ads_ more active), thus cleaning the Pt surface and increasing the number of metallic active sites available, for the subsequent hydrogen chemisorption, in the hydrogen region during anodic sweep of the second cycle.

CVs in the double-layer region (0.45–0.65 V) at different scan rate were carried out on Pt-DE to determine the double-layer capacitance (*C*_dl_)^46^ in the absence and in the presence of glucose, to prove its spontaneous chemical adsorption on the Pt surface. Figure 3a,b shows the CVs at different scan rates in the absence and in the presence of glucose in a PBS. Figure 3c shows the anodic and cathodic current density mean values at 0.55 V as a function of scan rate; from the slope, *C*_dl_ was calculated. It is worth noting how the *C*_dl_ values decreased from 33 ± 2 μF·cm^–2^ in PBS to 22 ± 1 μF·cm^–2^ when glucose was added, pointing to an adsorption of glucose molecules on the Pt surface, thus reducing the permittivity of the solution facing the electrode.

**Figure 3:**
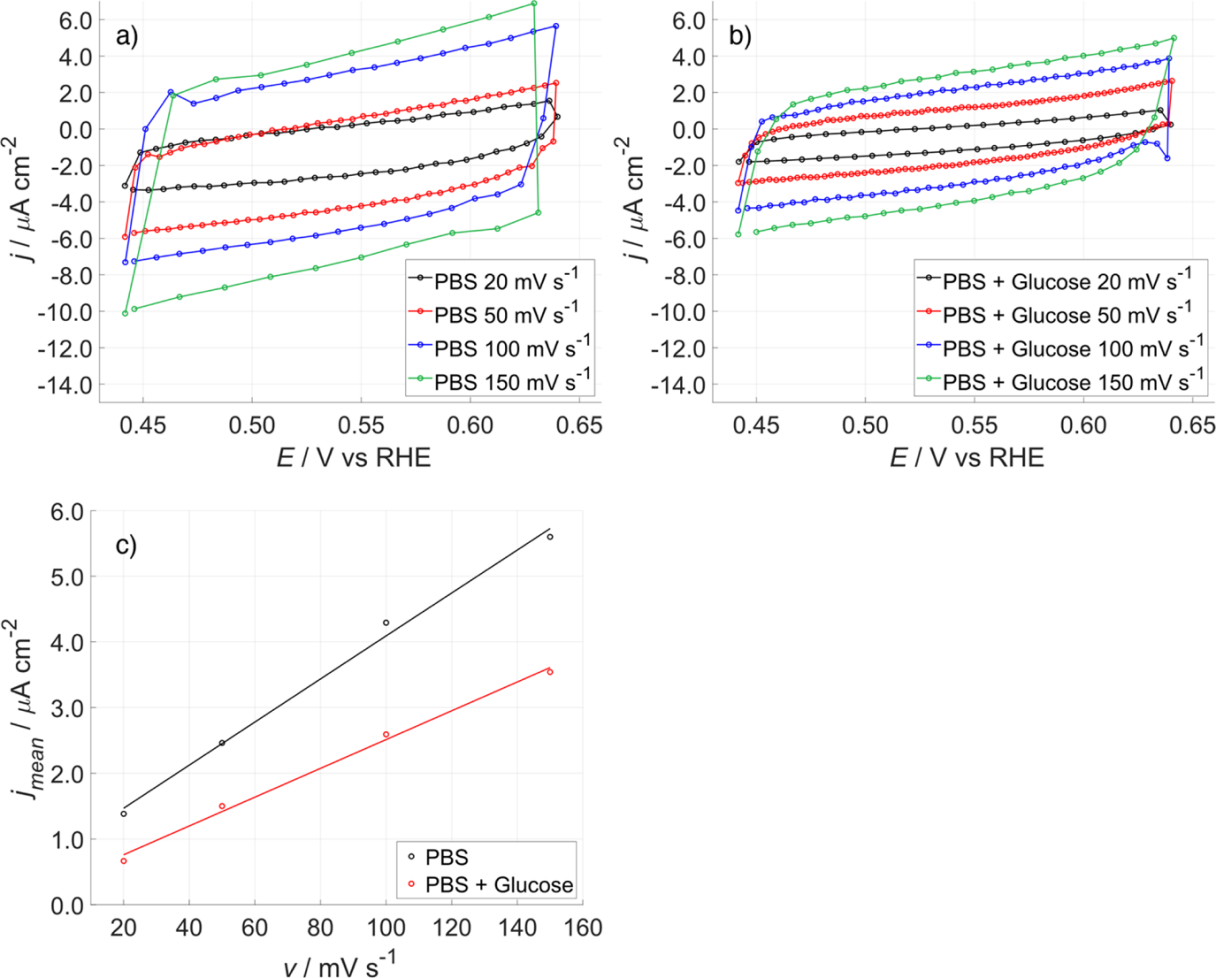
Double-layer capacitance measurement. CVs recorded at various scan rates in PBS (a) and PBS + 0.5 M glucose (b). (c) Plot of the current density vs the scan rate.

To better understand the glucose behavior in the three above described regions, CVs on Pt-DE in glucose + PBS were then carried out with the aim of exploring the relationship between glucose adsorption/dehydrogenation at potentials around 0.25 V, and glucose oxidation taking place in the “double-layer region and oxide region”. CVs were performed using an initial potential of 0.01 V and increasing the anodic final potential from 0.67 V up to 1.51 V; for each CV, 3 cycles were performed, see Figure 4.

**Figure 4:**
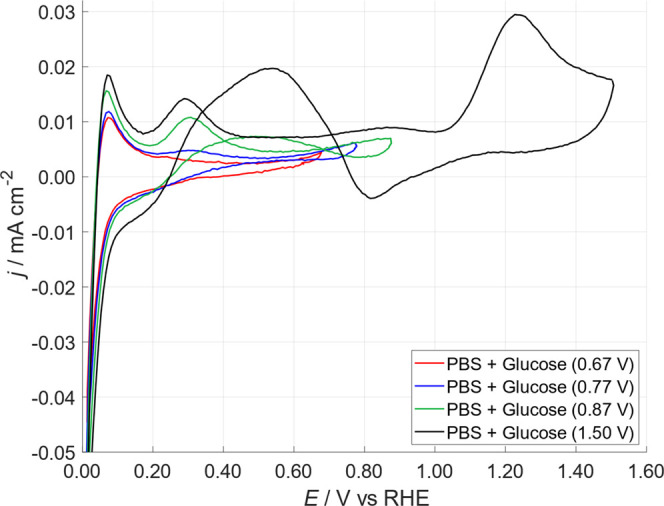
CVs on Pt-DE at increasing anodic final potential (see legend) in PBS + 0.5 M glucose: scan rate 50 mV·s^–1^. For the sake of clarity, only the third cycle is reported. All the potentials are referred to the reversible hydrogen electrode.

It is notable that the anodic peak around 0.25 V, attributed to glucose adsorption + dehydrogenation, was absent when the final potential was set to 0.67 V, while it clearly appeared when the latter was set at 0.87 V. We can speculate that, when the Pt surface was not polarized in the region of OH_ads_ formation, the glucose remained adsorbed on the Pt surface and the dehydrogenation of glucose, eq 2 describing peak 1 in Toghill and Compton,^28^ was suppressed in the third cycle, until the Pt surface was re-activated. This phenomenon also influenced the hydrogen oxidation peak at around 0.8 V, which increased as the anodic reversal potential was raised.
2

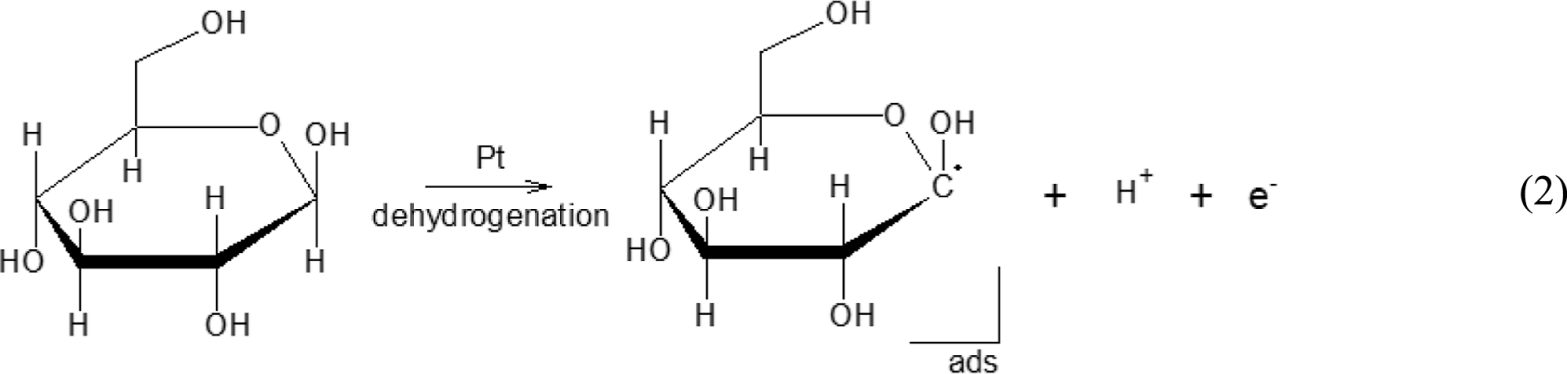




In fact, only when Pt reached potentials higher than 0.80 V did the glucose oxidation take place, thus leaving a clean Pt surface for the subsequent glucose adsorption and dehydrogenation.

### 3.2 Surface Interrogation-Scanning Electrochemical Microscope (SI-SECM)

The tip response in PBS with a ruthenium mediator was investigated using the setup explained in the experimental part. A first characterization was carried out immersing the tip in PBS, pH 7.3, in the presence of 1 mM [Ru­(NH_3_)_6_]^3+^ and scanning the potential between 0.23 and 0.63 V, see the black line in Figure 5, evidencing the expected response of a microelectrode in the bulk of a solution containing a reversible redox mediator. Black CV presents a not zero current, at *E* = 0.63 V, due to the presence of a small amount of oxygen, not removed by N_2_ due to the very small size of the cell (see Figure S2). Then, the tip was placed at about 3 μm from a non-polarized substrate, and the result is the red line in Figure 5. As described by Bard,^47^ the peak at 0.45 V vs RHE is due to the shift from positive to negative feedback when PtOx_ads_ are depleted. A third tip CV was acquired while maintaining the substrate polarized at +0.7, the blue line in Figure 5, which describes the condition of a constant amount of OH_ads_ generated on the substrate. Here, Ru^2+^ was produced at the tip and then immediately oxidized to Ru^3+^ on the substrate by the OH_ads_. The high tip current is explained by considering the continuous regeneration of Ru^3+^ at a short distance (short diffusion thickness) from the tip itself.

**Figure 5:**
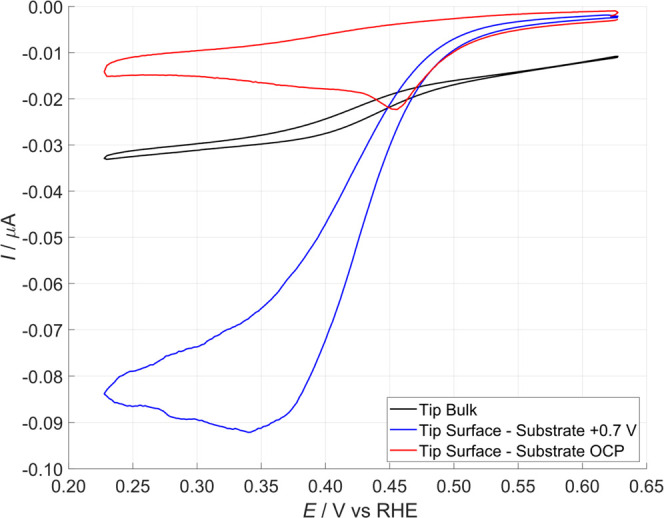
CV of Pt tip in PBS, pH 7.3, with 1 mM Ru­(NH_3_)_6_^3+^. Scan rate 50 mV·s^–1^. Black line: tip immersed in the solution. Red line: tip on the surface with negative feedback response. Blue line: tip on the surface with positive feedback response.

To investigate the effect of the Pt substrate polarization on the oxidizing species amount, we used the SI-SECM technique to interrogate the Pt surface after the substrate was polarized at different anodic potentials between 0.0 and 1.5 V vs RHE. These temporary substrate polarization potentials were labelled *E*_pre_ because they were applied only for 30 s before the analysis started and were switched off during the measurements. They were used to generate a selected amount of oxidizing species on the Pt substrate surface that were titrated by interrogating the substrate with the tip. In this analysis, as different values of these *E*_pre_ were applied at the substrate, we took also into account the direction, toward anodic or cathodic potentials, of variation of *E*_pre_. Briefly, the substrate was pre-polarized for 30 s at a selected constant potential (*E*_pre_), ranging from 0.01 to 1.51 V, and then from 1.51 to 0.01 V, the substrate polarization was then interrupted, and a CV was recorded at the tip to interrogate the substrate in the presence of [Ru­(NH_3_)_6_]^3+^. Figure 6 reports a typical tip voltammetry recorded after the substrate was polarized at 1.51 V in the absence and in the presence of glucose. In accordance with previous literature,^34,35^ the CV should exhibit a significantly high peak (typical of the SI-SECM operating in the feedback transient mode, as already seen in Figure 5, blue line) only during the cathodic scan of the first cycle. The peak area is in fact proportional to the amount of titrated PtOx_ads_ (red line Figure 6),^33,35^ and all the electrogenerated PtOx_ads_ was consumed during the first cathodic scan, leading to a much-decreased current in the second one. The only exception is related to a small peak at 0.43–0.47 V, observed also by Bard et al.^47^ and well described by the authors in the Supporting Information. This peak is due to the “residual oxygen” adsorbed on the Pt surface and formed, after the first cycle, for the presence of edge and defects.^33^

**Figure 6:**
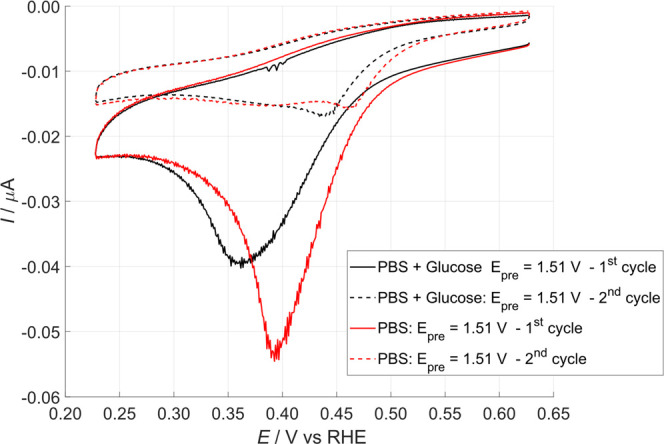
Surface interrogation of the substrate after polarizing the electrode at +1.51 V for 30 s in PBS, pH 7.3, with 1 mM of mediator couple, red line, and after the addition of 0.5 M glucose, black line. Scan rate 50 mV·s^–1^.

It is worth now to describe the behavior observed in the entire considered range of *E*_pre_ in the absence and presence of glucose.

Figure 7 reports tip CVs recorded after the substrate had been polarized for 30 s at various constant potentials, starting from 1.51 V and progressively shifting toward less anodic values with steps of 100 mV (negative direction). This approach resulted in varying amounts of oxidizing species (PtOx_ads_) formed on the substrate surface. When the substrate was polarized at the most anodic potential (1.51 V), the Pt surface accumulated the highest concentration of PtOx_ads_. Consequently, the mediator couple, which titrates these oxidizing species, generated the largest cathodic peak current. As the *E*_pre_ decreased, the amount of PtOx_ads_ formed was reduced, leading to a corresponding decrease in the cathodic peak current during the first CV cycle, see CVs in Figure 7a.

**Figure 7:**
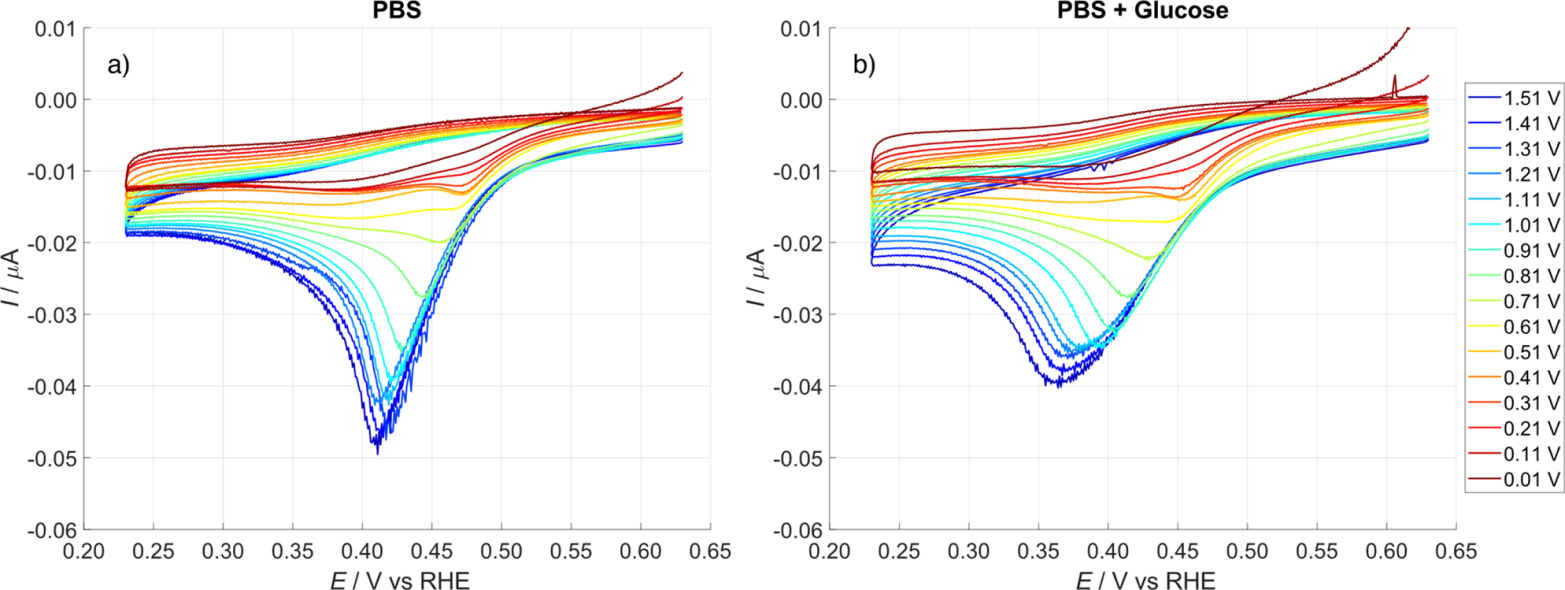
Titration of substrate oxidizing surface species formed at different substrate polarization constant potential (*E*_pre_), only the first cycle is shown. The investigation has been performed polarizing the substrate starting with the most anodic potential, 1.51 V, and moving towards less anodic potentials, up to 0.01 V (negative direction). Data for positive direction are reported in Supporting Information, Figure S7. (a) Electrolyte formed by PBS, pH 7.3, and 1 mM Ru­(NH_3_)_6_^3+^; (b) electrolyte containing also 0.5 M glucose. Scan rate 50 mV·s^–1^.

Notably, when *E*_pre_ exceeded 0.61 V, the CV shape changed significantly, showing a continuous increase in peak current due to the greater formation of oxidizing species on the Pt surface. This was accompanied by a light shift in the CV peak potential toward higher cathodic values.

The effect of glucose addition can be observed by comparing the data obtained for each *E*_pre_ value, as shown in Figure 7b. When the two CVs, recorded with and without glucose after substrate polarization at 1.51 V (see also Figure 6), were examined, the presence of glucose led to a decrease in the current peak. This decrement is attributed to the consumption of oxidizing Pt surface species by glucose oxidation, which in turn lowers the number of oxidants available to be titrated by the mediator couple. The charge associated with the first and second CV cathodic peak was calculated by integrating the respective current signals. The results are shown in Figure 8a for experiments carried out without and with glucose, respectively, for *E*_pre_ from 1.51 to 0.01 V versus RHE (negative direction). For the sake of clarity, only the integrated charges of the first cycle are reported because the charge values of the second cycle are almost zero, regardless of the applied *E*_pre_ value or scan direction. As mentioned, this indicates that available PtOx_ads_ on the Pt surface was nearly completely titrated during the first cycle.

**Figure 8:**
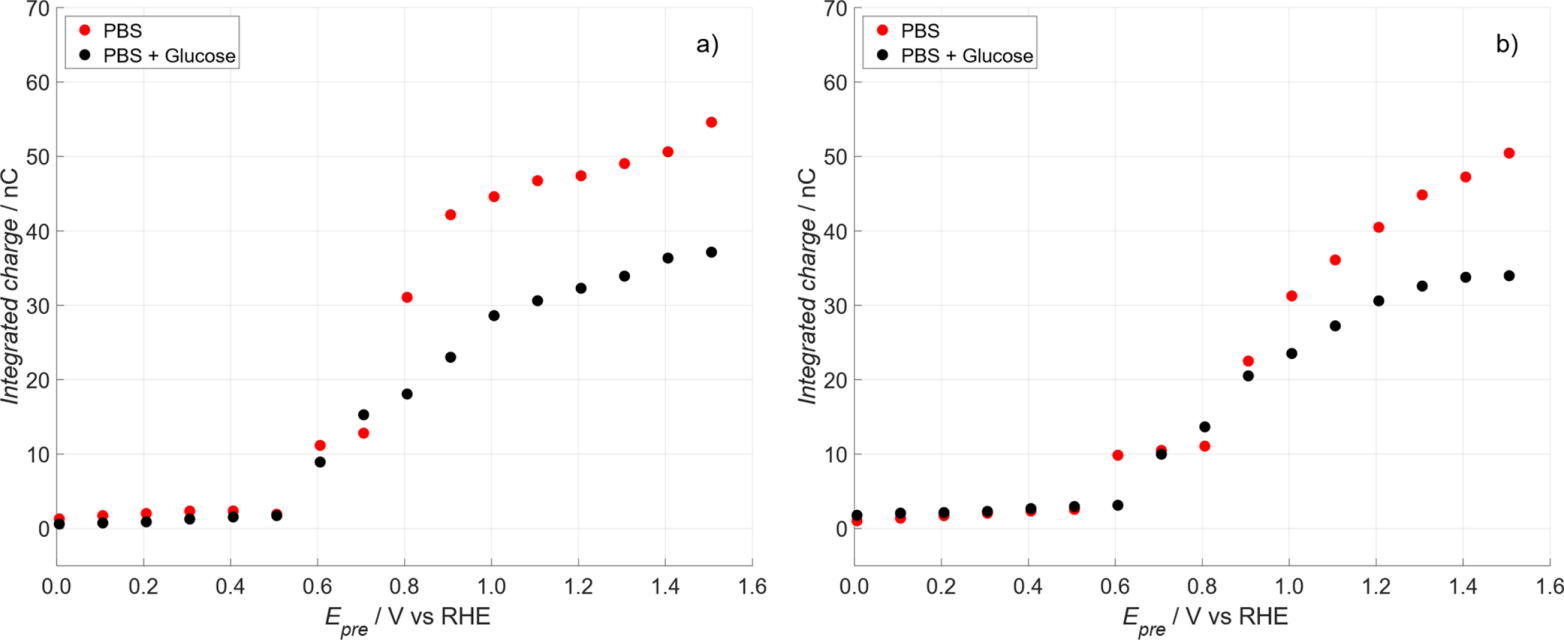
Comparison of the integrated charge of the first cycle obtained from the SI-SECM characterization. (a) *E*_pre_ from 1.51 to 0.01 V vs RHE (negative direction). (b) *E*_pre_ from 0.01 to 1.51 V vs RHE (positive direction). Black dots = without glucose. Red dots = with glucose. Electrolyte PBS, pH 7.3, with 1 mM Ru­(NH_3_)_6_^3+^. Each point represents the average of three measurements.

An analogous set of experiments was performed in the opposite direction; see Figure S6 in Chapter 2 of Supporting Information, scanning *E*_pre_ from 0.01 to 1.51 V vs RHE, Figure 8b. Data in Figure 8 are an average on 3 different experiments whose statistical analysis is reported in Table S1 (Supporting Information) as *p*-value of the *t*-Student test. *p*-values confirm the statistical difference between the two averages, at least for values above 0.81 V.

As shown in Figure 8, the formation of oxidizing species begins at approximately 0.61 V and increases progressively with more anodic *E*_pre_ values, independent of the presence of glucose or the *E*_pre_ polarization direction. Although the exact nature of the oxidizing species on the substrate surface remains unclear,^35^ it is generally accepted in the literature^28–30^ that within the double-layer region (0.61–0.8 V), OH_ads_ is the predominant species. When glucose was present, the peak current decreased for *E*_pre_ > 0.81 V in comparison with the experiments without glucose, due to the consumption of PtOx_ads_ by glucose oxidation, resulting in a lower integrated charge. This effect is clearly evidenced by comparing the trends of the red and black dots in Figure 8.

Assuming that the titrating agent Ru­(NH_3_)_6_^2+^ was consumed exclusively by platinum oxide species (PtOx_ads_), it can be inferred that the oxidizing species formed during the electrochemical process were rapidly consumed by glucose oxidation and were, therefore, no longer available for titration by the mediator. Moreover, based on the data in Figure 8, it can be speculated that the formation of PtOx_ads_ begins at potentials above 0.6 V, independently on the direction of substrate constant polarization potential (anodic or cathodic). However, the oxidizing species generated within the 0.61–0.81 V potential range do not appear to participate in glucose oxidation. This is evidenced by the similar responses observed in the presence and absence of glucose, as indicated by the overlapping data sets in the 0.61–0.81 V potential range of Figure 8a,b. Notable are the differences in the integrated charge between the glucose-free and glucose-containing experiment onsets at potentials above 0.7 V for the negative scan and above 0.9 V for the positive one, suggesting that only “high energy” PtOx_ads_ are involved in glucose oxidation. This behaviour is attributed to anion adsorption on the Pt surface, which alters the glucose oxidation mechanism, as previously described.^45,48^ This effect is more clearly illustrated in Figure 9, where the Pt-DE CV of Figure 2 is shown alongside data from SI-SECM titration of surface-bound oxidizing species. In the 0.5–0.8 V potential region, the amount of oxidized glucose, calculated by subtracting black from red dots of Figure 8 for both directions, is almost negligible, especially for positive potential direction; see the blue triangles.

**Figure 9:**
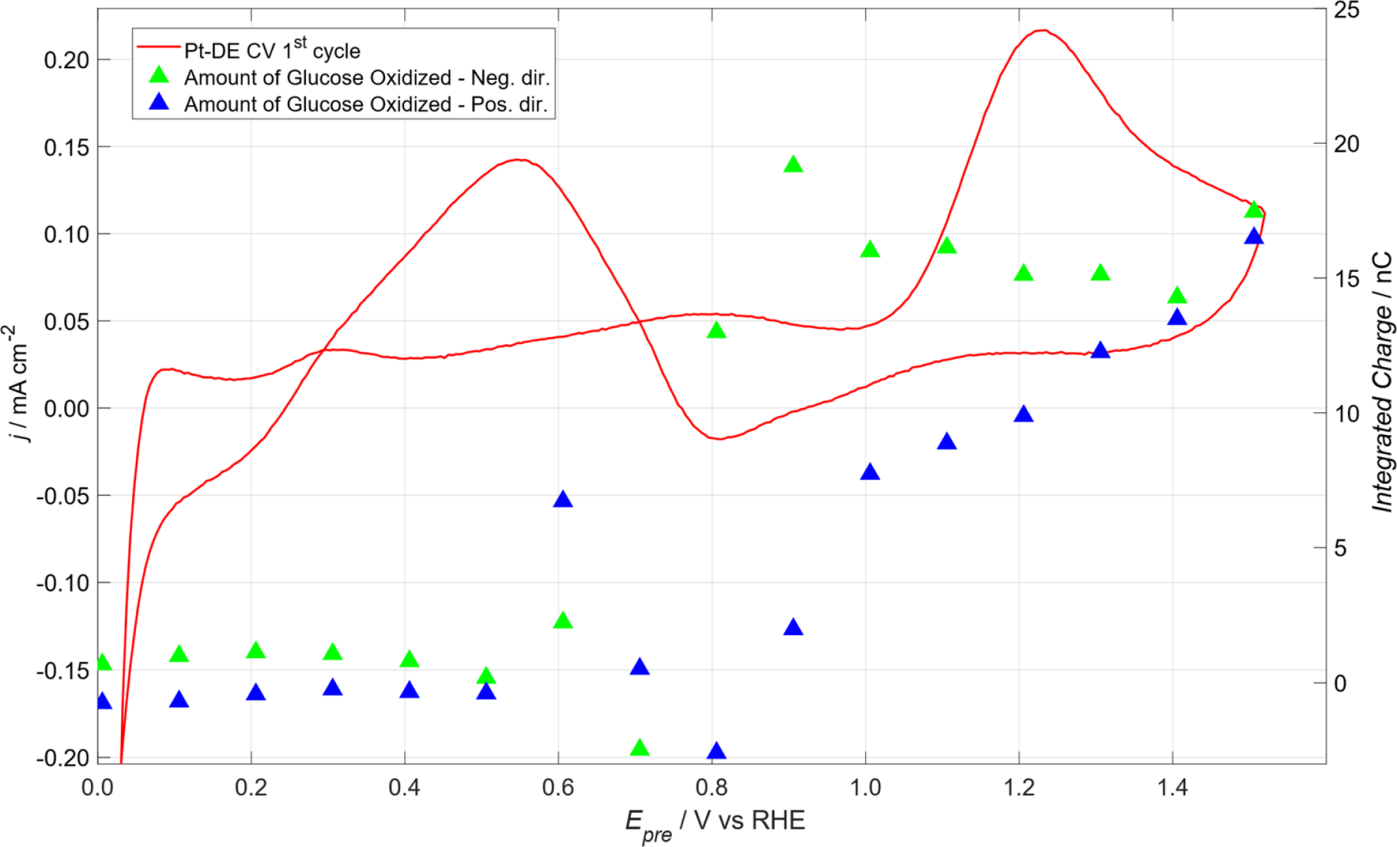
Red line: CV of Pt-DE presented in Figure 2, first cycle. Green triangle: amount of oxidized glucose obtained as difference between points of the first cycle of Figure 8a (red—black). Blue triangles: amount of oxidized glucose obtained as difference between points of the first cycle of Figure 8b (red—black).

It is worth highlighting the Raman-electrochemical study conducted by Huang et al.^49^ in acidic medium on Pt(111), which revealed the formation of OH_ads_ at potentials above 0.65 V. At potentials exceeding 1.0 V, shown in this work to be effective in glucose oxidation, the presence of platinum (su)­peroxide was also detected.

## 4 Conclusions

This study offers valuable insights into the electrooxidation of glucose on platinum electrodes in a neutral medium (PBS, pH 7.3), highlighting the role of platinum oxide species in the reaction, as revealed by the surface interrogation SECM technique. Investigations using a Pt macroelectrode confirmed the presence of three distinct regions in the anodic scan of the CV: the hydrogen region, the double-layer region, and the oxide region, each characterized by different surface-bound platinum oxidizing species. The CV data indicated that adsorbed glucose can be oxidized at potentials above 0.81 V, leaving active sites available for subsequent glucose adsorption. The initial adsorption and dehydrogenation of glucose were observed to occur at around 0.25 V.

The SI-SECM technique, employing a Ru­(NH_3_)_6_^3+^ mediator, enabled the titration of PtOx_ads_ species both in the presence and in the absence of glucose, evidencing also the role of anion adsorption in deactivating oxidizing species, specifically OH_ads_, in the double-layer region. This technique detects surface-bound platinum oxide moieties formed via anodic polarization. When applied in the presence of glucose, SI-SECM revealed a reduced amount of PtOx_ads_ on the substrate surface as part of these species were consumed during glucose oxidation.

The two potential regions described by Burke^29^ exhibit distinct behaviours with respect to oxidizing species, both of which are inactive or only partially active in glucose oxidation:

- Double-layer region (about 0.6–0.8 V): Pt oxide species, likely OH_ads_ as suggested by Burke, form in this range but are not consumed in glucose oxidation under our experimental conditions.
- Oxide region (above ca. 0.9 V): PtOx_ads_ species formed here are reactive and are consumed during glucose oxidation.

By combining CV and SI-SECM data, it is possible to map the sequence of events occurring during the anodic polarization of Pt in the presence of glucose, supporting the mechanistic framework proposed in the literature.^28^ Glucose adsorption and dehydrogenation occur around 0.25 V. From 0.61 V, OH_ads_ begins to form on the Pt surface; however, no glucose oxidation is observed at this stage. Oxidation begins above 0.81 V, consistent with findings from Pletcher and others.^26,29,30^ Overall, the experimental evidence indicates that glucose oxidation proceeds concurrently with the formation of PtOx_ads_ species, leading to their immediate consumption.

In conclusion, this investigation advances the field of non-enzymatic glucose sensing by elucidating key mechanistic factors that influence sensor performance. These insights can guide the development of more efficient, cost-effective, and reliable CGM technologies for diabetes management and broader bioelectrochemical applications.

## Supplementary Material



## Notes

Additional experimental details, materials, and methods, including photographs of experimental setup (PDF)
