# Supplementary material for: Non-enzymatic Glucose Detection Mechanism on Pt: A Surface Interrogation Scanning Electrochemical Microscopy Investigation
Source: ACS Electrochem. 2026 Apr 27;2(5):1138–47. doi: 10.1021/acselectrochem.5c00464 (PMC13159967; doi:10.1021/acselectrochem.5c00464)
Supplement: Supplementary file 1 [file ec5c00464_si_001.pdf]

# Supporting Information

# Non-enzymatic glucose detection mechanism on Pt: a surface interrogation scanning electrochemical microscopy investigation

Nazario Martino[1], Francesco Panico[1], Frank Marken[2], Alberto Vertova[*,1,3], Alessandro Minguzzi[1,3,4]

1 Dipartimento di Chimica, Università degli Studi di Milano, via Golgi 19, 20133 Milano, Italy.
2 Department of Chemistry, University of Bath, Bath BA2 7AY, United Kingdom
3 Consorzio Interuniversitario di Scienze e Tecnologia dei Materiali, Via San Giusti 9, 50121 Firenze, Italy.
4 Dipartimento di Energia, Politecnico di Milano, Via Lambruschini 4a, 20156 Milano, Italy

*Corresponding Author: alberto.vertova@unimi.it*

## Contents

1. Figure S1. Optical microscope image of plastic Pt tip use in SI-SECM measurements. White line: scale bar.
2. SECM – Positioning
    a. Figure S2, S3, S4 and S5
3. SI-SECM – Anodic Direction: Figure relevant to the anodic scan of the $E_{pre}$.
    a. Figure S6.
4. Table S1. *p*-value of t-Student statistical analysis

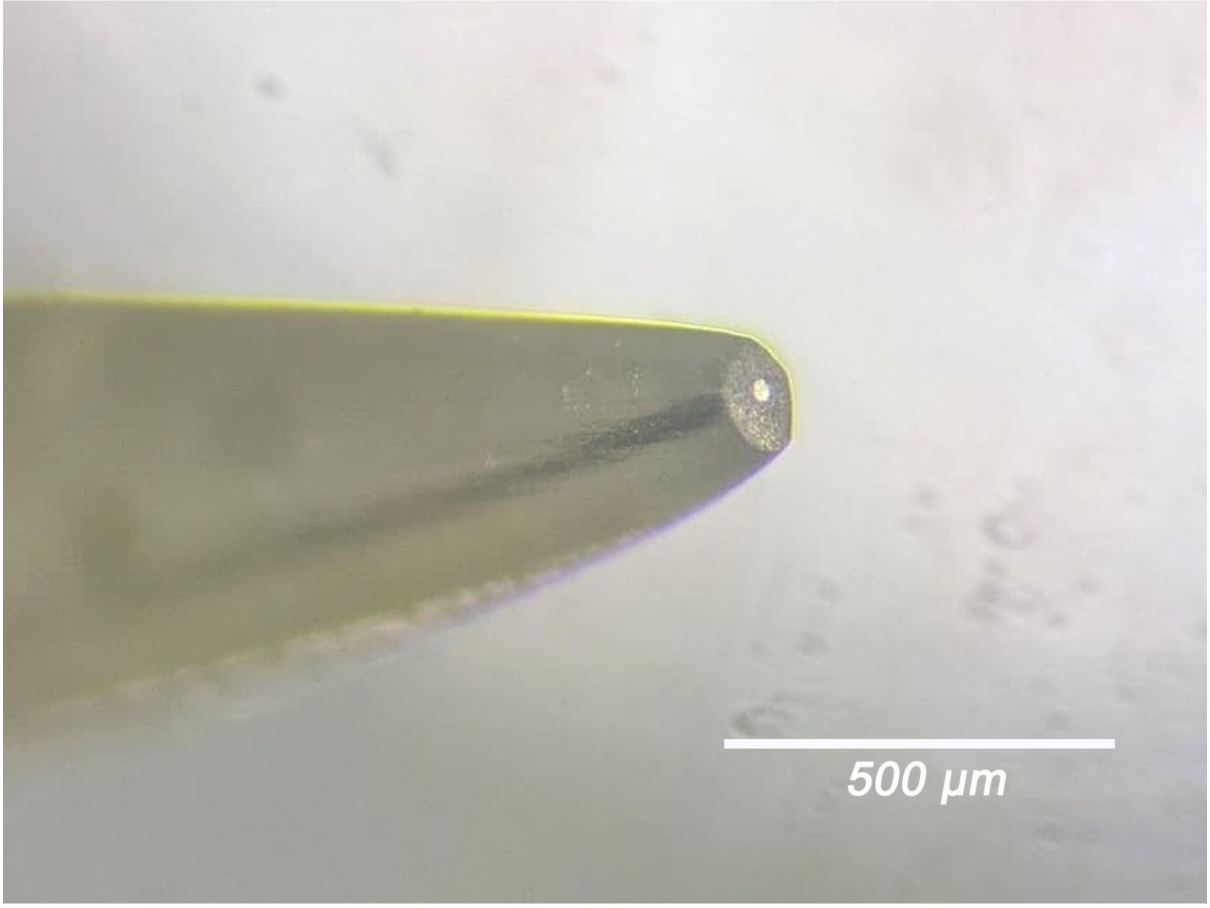


***Figure S1**. Optical microscope image of plastic Pt tip use in SI-SECM measurements. White line: scale bar.*

## 1. SECM – Positioning

The electrode setup, as shown in Figure S2, was achieved by inserting the substrate from below a drilled Teflon electrochemical cell and positioning the SECM tip above it.

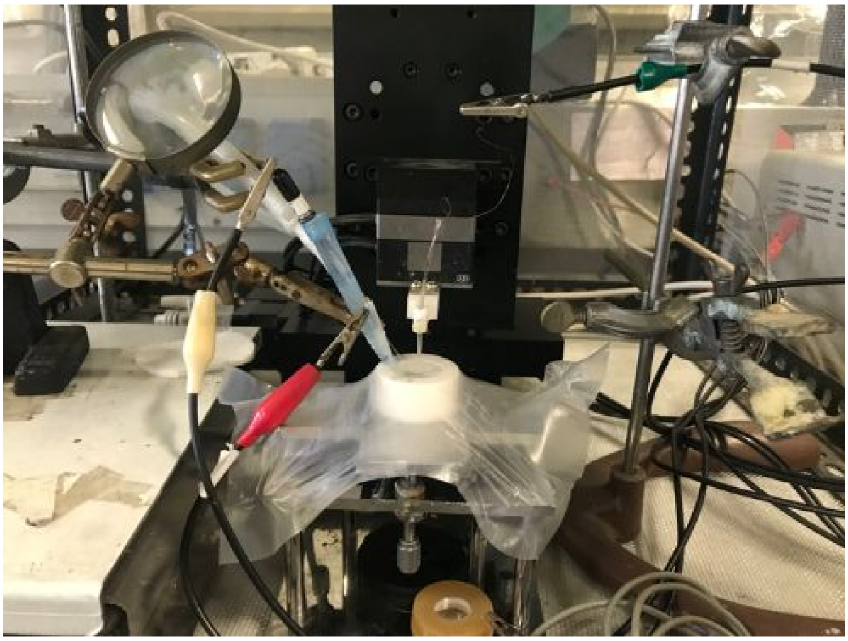

***Figure S2***. *SECM, system setup.*

The tip was moved to locate electrochemically the centre of the substrate; the alignment procedure is described below.

### *1.1 Approaching curve*

Two approaching curves (Figures S4*a* and *b*), carried out in feedback mode, with two different approaching speeds were registered with the following parameters:

- Fast
    - Probe E: 0.23 V vs RHE
    - X Distance ($\mu m$) = 900
    - Incr. Dist. ($\mu m$) = 0.4
    - Incr. Time (s) = 0.02
    - Quit time (s) = 0
- Slow
    - Probe E: 0.23 V vs RHE
    - X Distance ($\mu m$) = 900
    - Incr. Dist. ($\mu m$) = 0.02
    - Incr. Time (s) = 0.02
    - Quit time (s) = 0

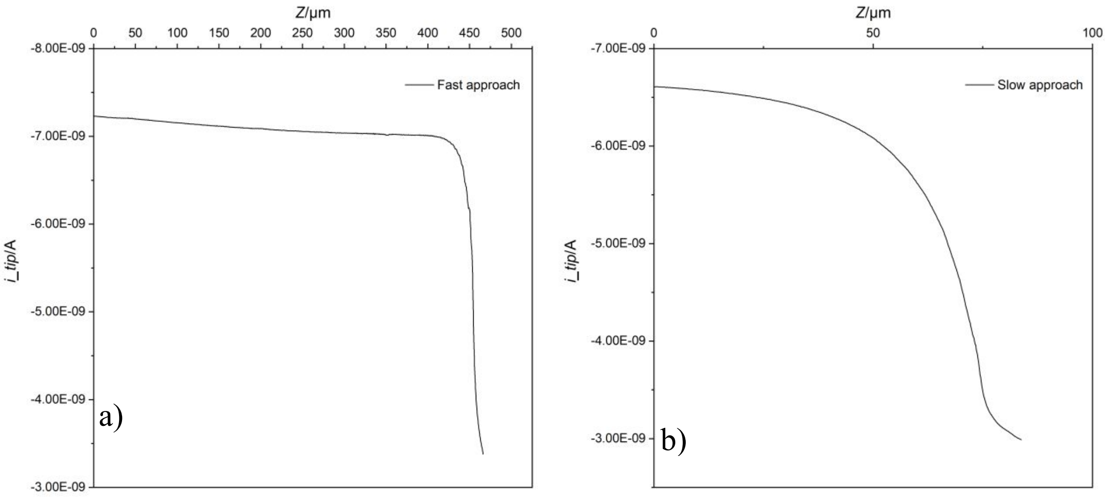


***Figure S3***. *Approaching curves in PBS 0.1 M pH=7.3 + 1 mM $Ru(NH_3)_6Cl_3$ after degassing with $N_2$. (a) fast approaching curves (b) slow approaching curves*

This procedure allows the operator to position the tip sufficiently close to the substrate to carry out the alignment also along the x and y axes, in the next step.

### *1.2 x, y alignment*

The relative positioning of the two electrodes is crucial in this experiment and is done first by means of the Generation/Collection mode, which is effective over longer distances and then by positive feedback, Figure S4

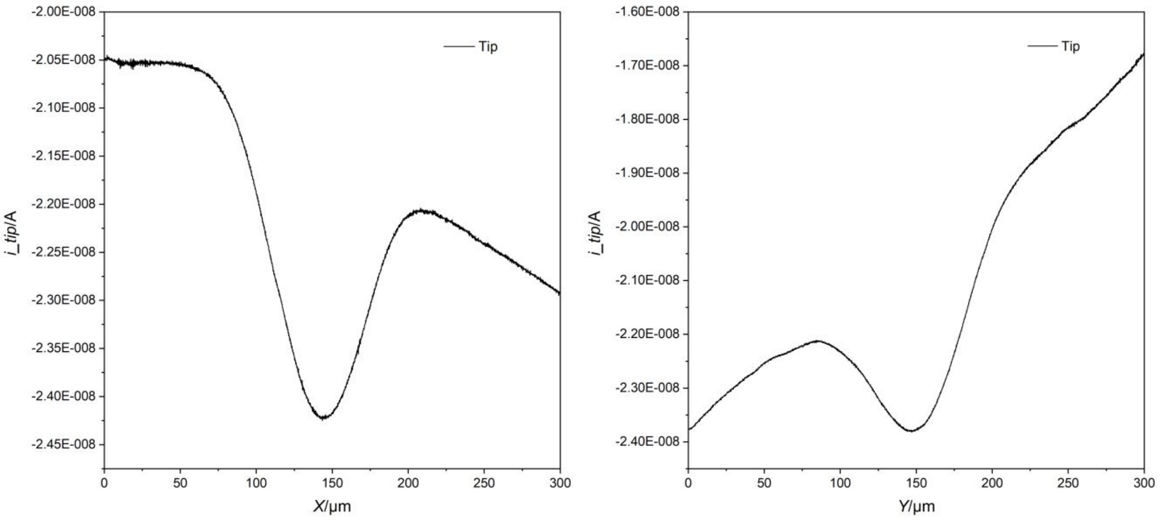


***Figure S4***. *x,y alignment feedback mode curves in PBS 0.1 M pH = 7.3 + 1 mM $Ru(NH_3)_6Cl_3$ after degassing with $N_2$*

### *1.3 SECM: individuation of the platinum wire substrate*

In order to individuate the Pt substrate, a SECM measurement in G/C mode is performed.

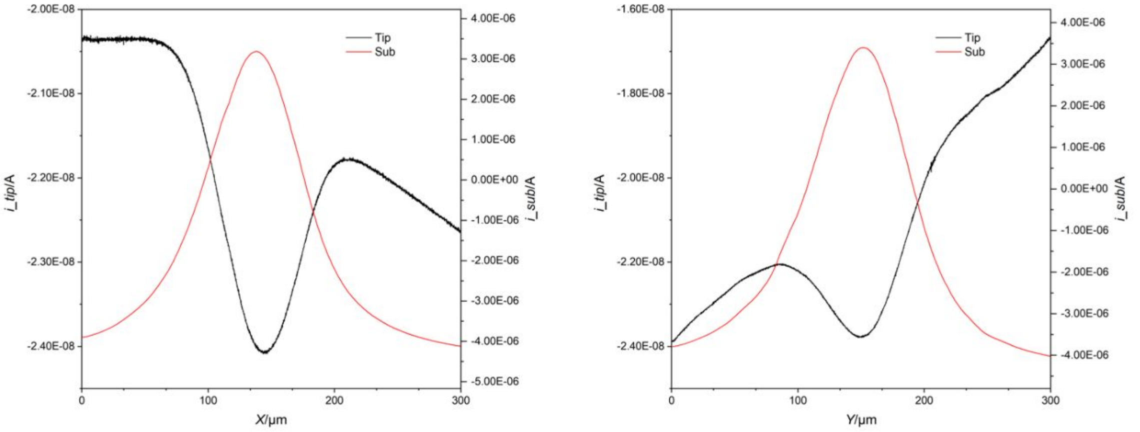


***Figure S5**. Lateral scans for locating the center of the substrate electrode, using the mediator. $Ru(NH_3)_6^{2+}$ is produced at the substrate, while the tip is oxidizing it back to $Ru(NH_3)_6^{3+}$.*

## 2. SI-SECM – Anodic Direction

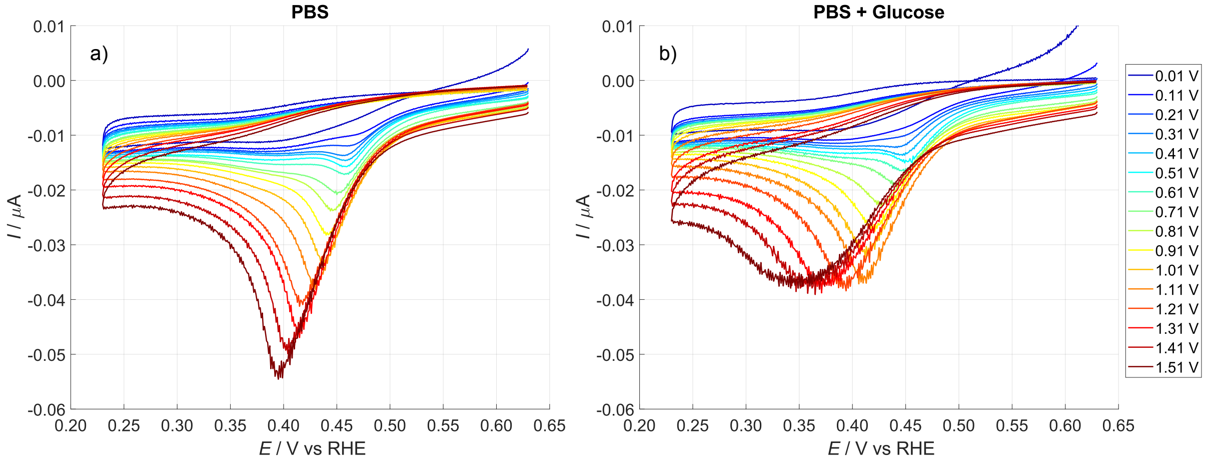


***Figure S6.*** *Titration of Pt-UME$_{sub}$ oxidizing surface species formed at different substrate polarisation constant potential ($E_{pre}$), only the first cycle is shown. The investigation has been performed polarizing the substrate starting with the most cathodic potential 0.01 V, and moving towards less cathodic potentials, up to 1.51 V (positive direction). a) solution containing PBS, pH 7.2 and 1 mM $Ru(NH3)_6^{3+}$, b) solution containing also 0.1 M glucose.*

**Table S1.** *p*-value of t-Student statistical analysis of data presented in **Figure 8.** At higher overpotentials the difference is significative.

| $E_{pre}$ | *p*-value | $E_{pre}$ | *p*-value |
|---|---|---|---|
| **0.01** | 0.506 | **1.51** | 0.070 |
| **0.11** | 0.500 | **1.41** | 0.091 |
| **0.21** | 0.666 | **1.31** | 0.083 |
| **0.31** | 0.819 | **1.21** | 0.120 |
| **0.41** | 0.728 | **1.11** | 0.141 |
| **0.51** | 0.637 | **1.01** | 0.160 |
| **0.61** | 0.410 | **0.91** | 0.168 |
| **0.71** | 0.913 | **0.81** | 0.286 |
| **0.81** | 0.898 | **0.71** | 0.818 |
| **0.91** | 0.823 | **0.61** | 0.827 |
| **1.01** | 0.322 | **0.51** | 0.846 |
| **1.11** | 0.346 | **0.41** | 0.449 |
| **1.21** | 0.274 | **0.31** | 0.208 |
| **1.31** | 0.146 | **0.21** | 0.029 |
| **1.41** | 0.047 | **0.11** | 0.002 |
| **1.51** | 0.018 | **0.01** | 0.252 |
